# ADP-ribosylation factor 6 expression increase in oesophageal adenocarcinoma suggests a potential biomarker role for it

**DOI:** 10.1371/journal.pone.0263845

**Published:** 2022-02-10

**Authors:** Venkateswarlu Kanamarlapudi, Salman Tamaddon-Jahromi, Kate Murphy

**Affiliations:** 1 Institute of Life Science 1, School of Medicine, Swansea University, Singleton Park, Swansea, United Kingdom; 2 Cellular Pathology, Swansea Bay University Health Board, Singleton Hospital, Swansea, United Kingdom; National Cancer Institute, UNITED STATES

## Abstract

ADP-ribosylation factor 6 small GTPase plays an important role in cell migration, invasion and angiogenesis, which are the hallmarks of cancer. Although alterations in ARF6 expression and activity have been linked to metastatic cancer in one or two tissues, the expression of ARF6 in cancers over a wide range of tissues has not been studied so far. In this report, we analysed the expression of *ARF6* mRNA in cancers and corresponding healthy controls from 17 different tissues by real-time qualitative polymerase chain reaction (RT-qPCR). We further evaluated ARF6 protein expression in oesophageal adenocarcinoma (EAC) tissue microarray cores by immunohistochemistry. The ARF6 gene expression levels are highly variable between healthy and cancer tissues. Our findings suggest that the *ARF6* gene expression is up-regulated highest in oesophageal cancer. In EAC TMAs, ARF6 protein expression increase correlated with EAC progression. This is the first study to investigate *ARF6* gene expression in a wide array of cancer tissues and demonstrate that *ARF6* expression, at both mRNA and protein levels, is significantly upregulated in higher grades of EAC, which may be useful in targeting ARF6 for cancer diagnostic and therapeutic purposes.

## Introduction

Cancer is one of the leading causes of death in the world. In 2018, there were an estimated 18.1 million new cases and 9.6 million cancer-related deaths worldwide [1]. Despite advances in early detection and effective treatments, globally cancer incidents are predicted to increase to >23 million annually by 2030 and its mortality rate has also risen by 25% since the 1990s. The increase in cancer mortality rate and incidents is due to a possible combination of various factors such as exposure to carcinogens, unhealthy lifestyle choices, age, inflammation and a genetic predisposition from acquired or inherited polymorphisms [2–4]. The increased number of people living with cancer provides a mandate for developing new therapeutics and diagnosis methods for cancer.

Cancer is defined as an uncontrolled growth that is resistant to antigrowth signals, evades apoptosis, has unlimited replicative potential, can stimulate angiogenesis, and has invasion/metastatic potential [5]. During metastasis, cancer cells invade and pass through the lymphatic or circulatory system. The metastatic cancer cells can, therefore, reside at sites away from the primary tumour and form secondary tumours. Several signalling molecules including the Ras superfamily of small GTPases have been suggested to play important roles in cancer development and progression [6]. Consistent with this, alterations in activity and expression of the Ras superfamily GTPases have been reported in various cancers [7–9].

Small GTPases of the Ras superfamily are ~21kDa in size and cycle between the GTP-bound active and GDP-bound inactive conformations. In the active conformation, they bind to their effectors and induce downstream signalling. In the inactive conformation, the Ras superfamily GTPases cause signalling to cease. In general, small GTPases are activated by guanine nucleotide exchange factors (GEFs), which catalyse the exchange of GTPase bound GDP to GTP, and inactivated by GTPase activating proteins (GAPs), which induce hydrolysis of the GTPase bound GTP to GDP [10]. Based on sequence and functional similarities, the Ras superfamily is sub-divided into five families (Ras [Rat Sarcoma], Rho [Ras homologue], Rab [Ras-like protein from rat brain], Rad [Ras associated with diabetes], and ARF [ADP-ribosylation factor])–where their distinct cellular localisation (determined by their ‘active’ and ‘inactive’ state) and tissue distribution infers their diverse functional roles [10]. The ARF family small GTPases are further divided into 3 classes. The classes I (ARFs 1–3) and II (ARFs 4 and 5) Arfs localise mainly to the Golgi where they are thought to participate in membrane trafficking events, although some evidence has emerged for the activity of ARF1 and ARF4 at the plasma membrane [11, 12]. The sole member of class III ARFs, ARF6, cycles between the plasma membrane and the endosomal compartment where it coordinates membrane trafficking and cytoskeletal reorganisation [13, 14].

The families of ARF GEFs which show specificity for ARF6 are the ARNOs/cytohesins [15, 16], the EFA6s [17] and the BRAGs [18–20]. The effectors of ARF6 include phospholipase D (PLD), phosphatidylinositol 4-phosphate (PI4P) 5 kinase (PIP5K) and JIP3/4. PLD converts phosphatidylcholine (PC) to phosphatidic acid (PA), which plays a role in exocytosis from adrenal chromaffin cells [21], endosomal recycling [22]. It has also been implicated in ARF6-mediated H-Ras induced transformation [23]. PIP5K phosphorylates PI(4)P to PI 4,5-bisphosphate (PI(4,5)P_2_), which has been linked to clathrin-mediated endocytosis (CME), exocytosis of neurotransmitters and insulin, and endosomal recycling [24]. Given that PI(4,5)P_2_ is also required for PLD2 activation [25] and PA stimulates PIP5K [26], it is perhaps unsurprising that ARF6 has been implicated in cellular events such as exocytosis and endosomal recycling that are regulated by both these enzymes. JIP3 and JIP4 proteins allow ARF6 to traffic along microtubules during cytokinesis [27] and also enable fast recycling of cargo following CME [28].

ARF6 expression is altered in breast, glioma and lung cancers [29–31]. In this report, a more widespread analysis of *ARF6* gene expression in healthy and cancer tissues was sought using the Origene’s TissueScan Cancer Survey cDNA array (381 samples covering 17 different cancers). Although the expression of *ARF6* in some cancers has been investigated previously, this is the first time a widespread array of cancer tissues has been used to investigate the expression of this gene. This analysis revealed a significant increase in ARF6 mRNA expression in oesophageal cancer, which is confirmed by analysing ARF6 protein expression in oesophageal adenocarcinoma (EAC) tissue microarray cores by immunohistochemistry using an anti-ARF6 antibody.

## Methods

### Polymerase chain reaction (PCR)

The specificity of real-time qualitative PCR (RT-qPCR) primers was validated using a standard PCR [32]. The PCR reaction was performed in a final volume of 25μl containing 1 unit Red Taq DNA polymerase (Sigma), 0.2mM dNTPs, 0.2μM primers and 10ng of plasmid DNA (ARF6/pEGFP-N1 or ARF1/pEGFP-N1). The 5′ (sense) and 3′ (antisense) primers used for ARF6 PCR were 5′-TGTGGGTTTCAACGTGGAGAC-3′ and 5′-CAGTGTAGTAATGCCGCCAGAG-3′. The amplification was carried out by an initial denaturation at 94°C for 2 min followed by 30 cycles of denaturation at 94°C for 30sec, annealing at 60°C for 30sec and extension at 72°C for 1min. The reaction was terminated by a 5min extension step at 72°C. The PCR products were run on a 1.5% agarose gel, which was prepared in Tris Acetate EDTA (TAE) containing 1μg/ml ethidium bromide, at 100 volts in TAE buffer for 30min, and the gel was then visualised using a GelDoc (BioRad).

### RT-qPCR

The cycle threshold values were determined by performing RT-qPCR using an eight-fold dilution series starting with 1ng ARF6/pEGFP-N1 plasmid DNA or 10ng MDA-MB-231 cDNA. A graph was plotted with Log_10_ of the DNA concentration on the X-axis and Ct on the Y-axis to analyse cycle threshold values. The reaction efficiencies (E) were calculated by 10^−1/gradient^ and percentage efficiency was derived through the equation %E = E^-1^ x 100. Origene cancer survey cDNA array (381 samples covering 17 different cancers), which is normalised with β-actin, was used for differential *ARF6* gene expression analysis by RT-qPCR. RT-qPCR was carried out with SensiMixPlus SYBR & fluorescein kit (Quantace, London, UK) as per the manufacturer’s instructions. Briefly, RT-qPCR reactions (25μl) were carried out in a 96-well PCR plate using a MyIQ (BioRad) thermocycler and the following protocol: enzymatic activation at 95°C for 10min, followed by 45 cycles of denaturing of the DNA at 95°C for 15sec, annealing of the primers at 60°C for 30sec and 72°C extension step for 30sec. The relative fold change in expression was calculated by using the 2^-ΔΔCt^ method, where ΔΔCt is equal to the difference between ΔCt of sample and ΔCt of reference, and ΔCt is the Ct value for sample or reference normalised to the endogenous housekeeping gene (β-actin) [33].

### Immunohistochemistry

Immunohistochemistry (IHC) was performed using BenchMark ULTRA automated immunostainer (Roche-Ventana, US). The tissue microarray (Biomax, US; Cat. No. ES8011a), containing 35 cases of EAC and 5 healthy tissues in duplicate, were incubated in CC1 retrieval buffer (pH8-8.5) for 40 minutes, previously validated anti-ARF6 3A-1 mouse monoclonal antibody (Santa Cruz Biotech., US) at a dilution of 1:150 was applied and incubated at 36°C for 40min [32, 34–36]. The specificity of the ARF6 antibody was also confirmed by immunoblotting (**S1 Fig**). OptiView HQ universal linker and HRP multimer were added for 8min to enhance stain quality. Diaminobenzidine (DAB) was used as the chromogen, and samples were counterstained with hematoxylin for 12min. Tissues were scored based on the proportion of the epithelial cells that showed staining and the intensity of the stain.

### Statistical analysis

The Mann-Whitney statistical test was used to calculate statistical significance for two unpaired groups, while the Kruskal -Wallis test was used to compare three or more unmatched groups. Both of these tests are nonparametric and therefore they make fewer assumptions about the distribution of the data. Statistical significance was defined as a p-value of ≤0.05, signifying a 5% or lower probability of the data occurring by chance. However, p-values ≤0.01 and ≤0.001 were defined as highly statistically significant and very highly statistically significant, respectively.

## Results

### Validation of primers for ARF6 and determining cycle threshold values by RT-qPCR

**Fig 1:**
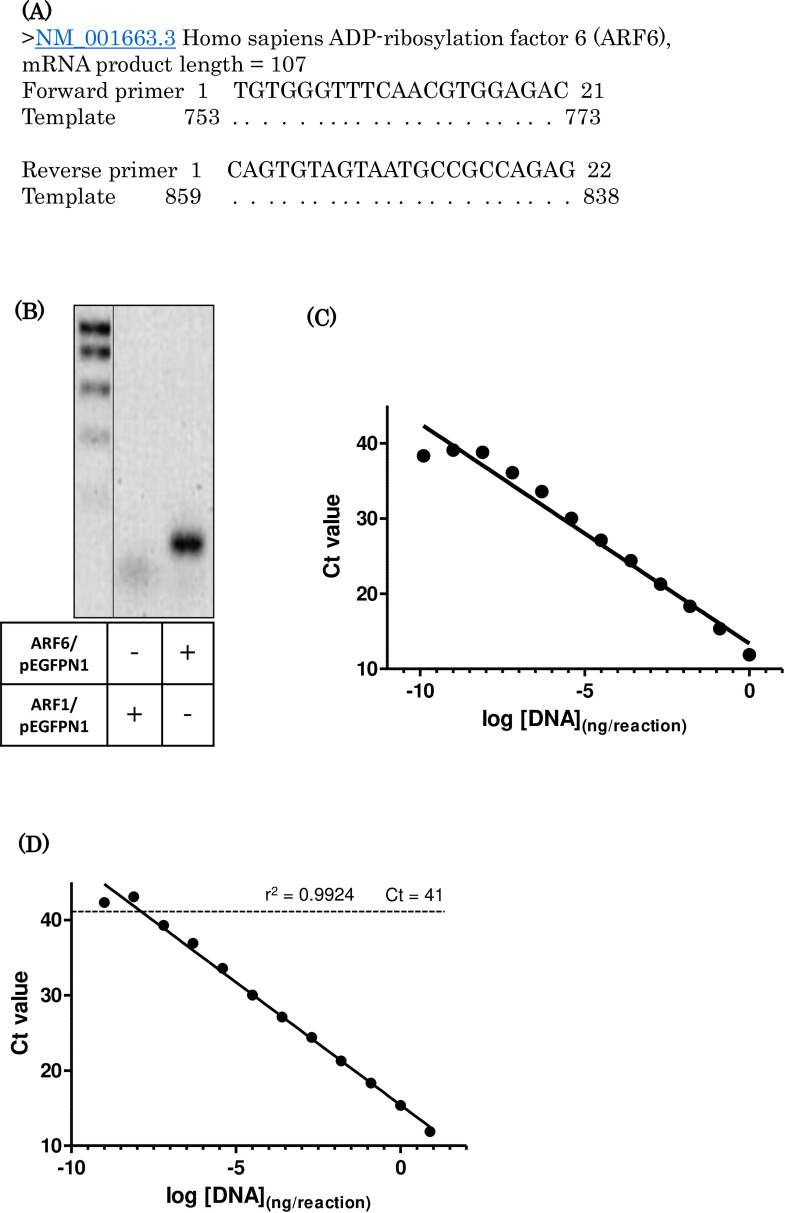
Validation of the ARF6 primers by using RT-PCR and obtaining cycle threshold values by performing RT-qPCR. (A) Alignment of ARF6 primer sequence with ARF6 gene sequence to estimate the ARF6 amplicon size. B) PCR products showing the specificity of ARF6 primers. (B) Agarose gel electrophoresis of PCR products obtained using the ARF6 primers with either ARF6/pEGFP-N1 or Arf1/pEGFP-N1 plasmid as a template (ladder: 1000 bp; 800 bp; 600 bp; 400 bp; 200bp). Cycle threshold values were obtained by performing ARF6 RT-qPCR using a dilution series of ARF6 encoding plasmid DNA (C) or MDM-MB-231 cDNA (D).

The ARF6 RT-qPCR primers used in this study were first validated by assessing the specificity using conventional RT-PCR and efficiency by RT-qPCR (**Fig 1**). **Fig 1A** is a readout from Primer-Blast, which compares the sequence of primers to the human genome. This shows that both the forward and reverse primers are identical to sections of the *ARF6* mRNA sequence (as signified by the dots beneath each base of the primer sequence) and are therefore specific for the amplification of this gene. The predicted size for the PCR product was calculated to be 107bp, which is within the <150bp criterion required to be suitable for RT-qPCR. A PCR product of the expected size was obtained with the ARF6 primers when ARF6/pEGFP-N1 plasmid DNA, but not closely related ARF1/pEGFP-N1, was used as a template, demonstrating the specificity of ARF6 primers (**Fig 1B**).

A dilution series of plasmid DNA encoding the ARF6 was used in RT-qPCR to study the minimum ARF6 cDNA copies that can still be detected (**Fig 1C**). A standard curve plotted from the results showed that assay runs linearly across the whole dilution series and as little as 7.5fg (10 copies) of plasmid DNA per reaction could still be detected by the RT-qPCR. An RT-qPCR was also performed using an 8-fold dilution series of MDA-MA-231 cDNA to define the range of Ct values within which ARF6 mRNA expression levels can be adequately determined (**Fig 1D**). This result revealed that the detection threshold of the assay is a Ct value of approximately 41. The PCR product obtained with MDA-MB-231 cDNA was sequenced to confirm that it is ARF6 (**S2 Fig**)

### Expression analysis of ARF6 in healthy tissues and cancers

Although there are several studies on linking ARF6 to cancer [29–31], particularly in invasion and migration behaviours crucial for metastasis, there is a dearth of data concerning the expression of *ARF6* in cancers over a wide range of tissue. To rectify this deficit, the expression of *ARF6* was analysed using the TissueScan Cancer Survey III panel (a collection of four 96-well plates loaded with cDNAs, pre-normalised to β-actin expression, from cancers and corresponding healthy controls from 17 different tissues) obtained from Origene.

**Fig 2:**
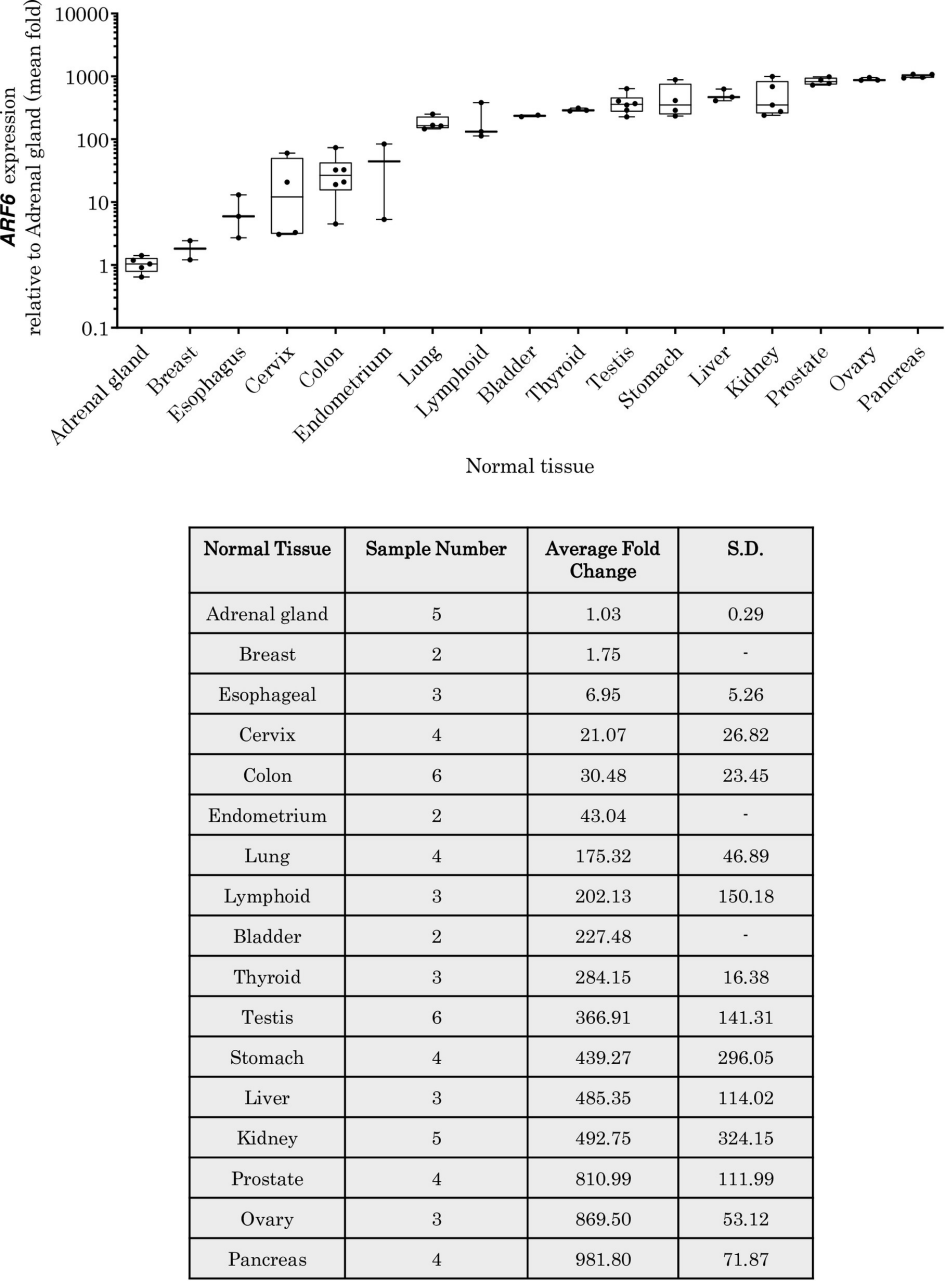
*ARF6* mRNA expression in human healthy tissues. The expression of ARF6 mRNA in healthy tissues relative to that in the adrenal gland (the lowest expressing tissue). Data is shown using a box plot with whiskers showing + SD.

*ARF6* mRNA expression was observed in all healthy human tissues used in this study, although the expression did vary from tissue to tissue (**Fig 2**). The lowest expression of *ARF6* mRNA was found in the adrenal gland whereas the highest expression was seen in the pancreas. The *ARF6* mRNA expression in healthy tissues can fall into four groups: very low expression (<100-fold that of adrenal gland expression) in the adrenal gland, breast, cervix, oesophagus, colon and the endometrium; low expression (<300-fold that of adrenal gland expression) in the lung, lymphatic tissue, bladder and thyroid; moderate expression (<500-fold) in the testis, liver, kidney and stomach; and high expression (>500-fold) in the prostate, ovary and pancreas.

**Fig 3:**
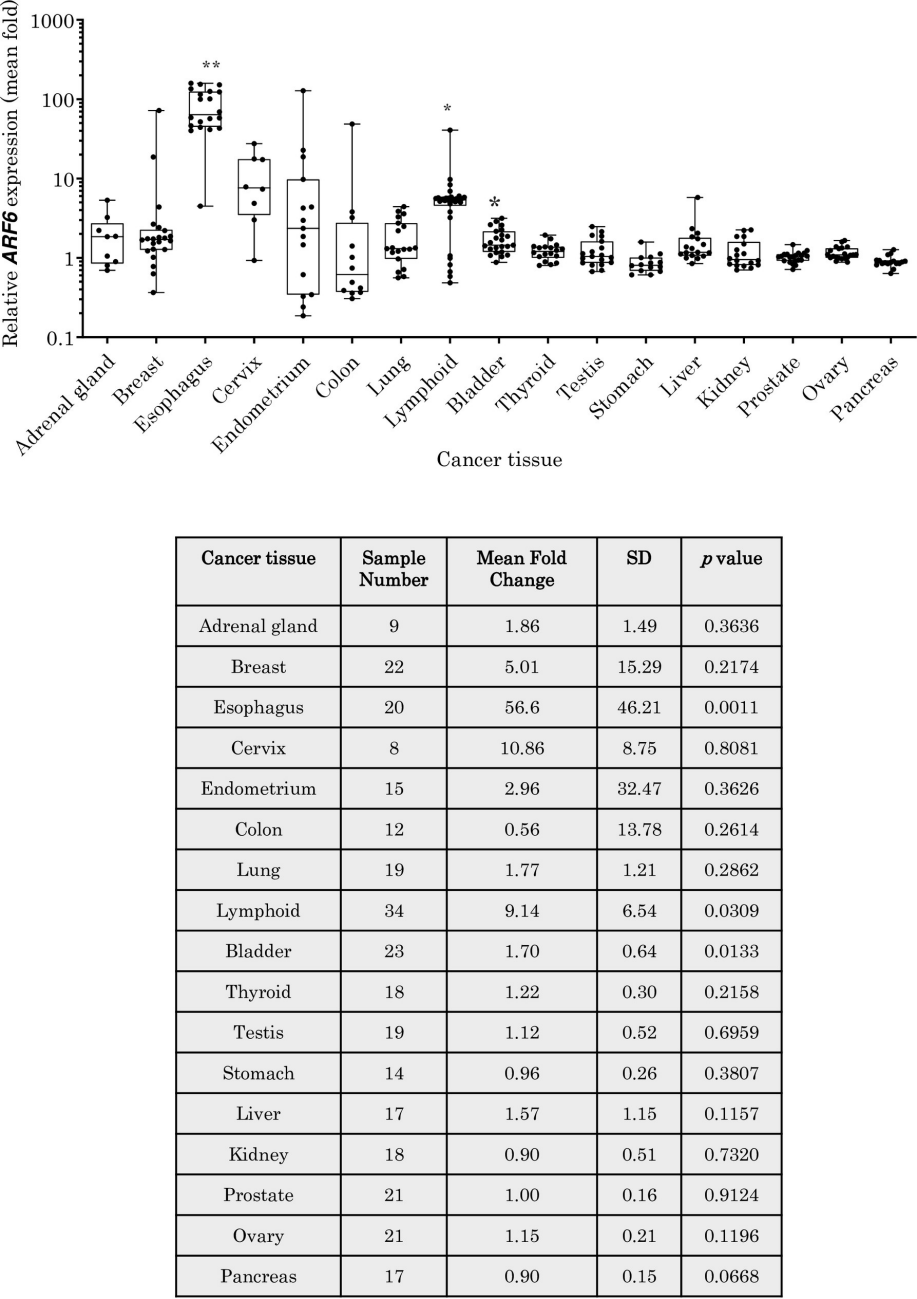
The expression of *ARF6* mRNA in human cancer tissues. Relative folds change in expression of *ARF6* mRNA in cancerous tissues when compared to that in healthy corresponding tissues. Data is shown using a box plot with whiskers showing + SD.

*ARF6* mRNA expression was then compared between the same tissues from healthy and cancer patients (**Fig 3**). There was very little difference in expression of *ARF6* at mRNA level between healthy and cancer tissues of the adrenal, colon, kidney, pancreas, stomach, prostate, ovary, liver, testis, thyroid, bladder, lung, cervix and endometrium, whereas alterations in the *ARF6* mRNA expression was observed in other cancer tissues studied. *ARF6* mRNA expression was shown to be up-regulated approximately 5-fold in breast cancer (statistically not significant; p = 02174), 9-fold in lymphoma (statistically significantly p = 0.0309) and 57-fold in EAC (statistically significant; p = 0.0011).

### *ARF6* mRNA expression increases with cancer progression in EAC

**Fig 4:**
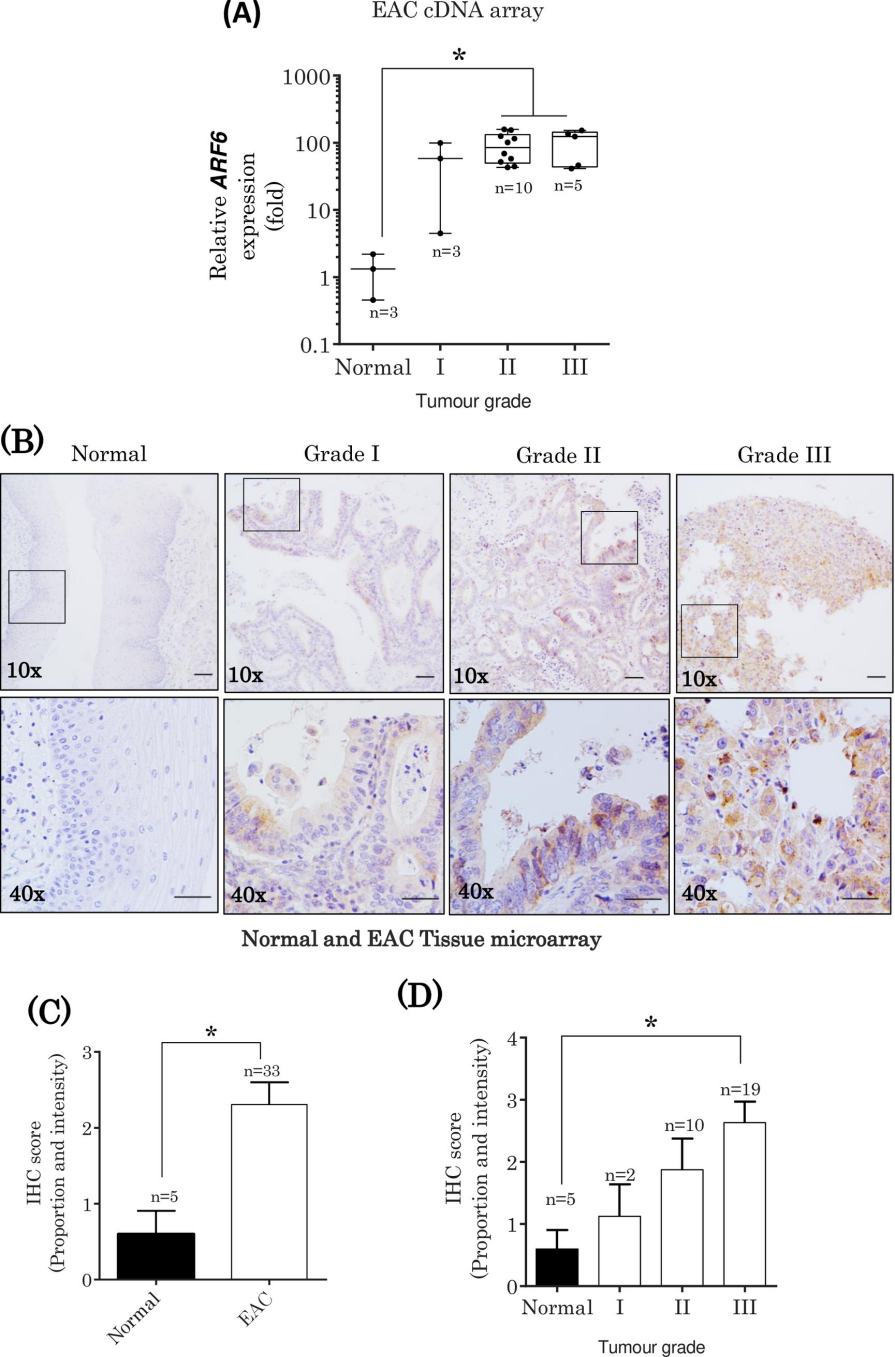
ARF6 protein expression increase in EAC. (A) Cancer stage-dependent expression of ARF6 mRNA in the oesophagus. (B) Immunostaining of EAC TMA was performed in a benchmark Ultra IHC staining module with an anti-ARF6 antibody. (C) The cores are displayed as 10x magnification and the insets were enlarged to 40x magnification. (D) Pooled ARF6 expression score between healthy and cancer specimens. (D) The cancer specimens were then separated into grades. Each core was scored based on the total sum of proportional of epithelial cells stained (score of 0 to +5) and the intensity of the staining (0 = none, +1 = weak, +2 = moderate and 3+ = strong). Scale bar 100μm.

The alternations in *ARF6* mRNA expression in EAC was explored further by separating the expression data by the grade of cancer (**Fig 4A**). Grading was assessed using AJCC guidelines that depend on the amount of cell abnormality. Cancer grading is a qualitative measure of cancer progression where grade I tumours are well-differentiated, grade II tumours are moderately differentiated and grades III-IV are poorly differentiated. Grades I-II usually reflect localised (benign) and locally invasive tumours whereas high-grade cancers (III-IV) denote invasion to neighbouring lump nodes and tissues and subsequent widespread metastasis to distant tissues. There is an up-regulation of *ARF6* mRNA expression in grade I EAC (average of 37 fold increase), but statistically not significant (p = 0.4433). *ARF6* mRNA expression is further increased in higher cancer grades II and III (average of 62- and 67-fold increase) showing statistical significance of p = 0.0197 and p = 0.0403, respectively.

### ARF6 protein expression increase correlates with an increase in EAC grading

Following the correlation found in *ARF6* mRNA expression increase in the EAC cDNA array, we also assessed changes in ARF6 protein expression in an EAC TMA by IHC (**Fig 4B**). ARF6 protein expression analysed using IHC was scored based on the proportion and intensity of ARF6 protein staining (**Fig 4B**). A visible increase in ARF6 protein staining was observed between healthy and EAC cores (p = 0.0233) (**Fig 4C**). Similarly, when the EAC cores were separated based on tumour grade, an increase in ARF6 expression was seen to correlate with tumour grade; reaching significant levels in grades II (although not statistically significant; p = 0.3415) and III (p = 0.0122) **(Fig 4D)**. Overall, we showed that ARF6 expression is upregulated during EAC cancer progression, highlighting this small GTPase as a potential biomarker for EAC.

## Discussion

Although alterations in the expression levels of ARF6 have been reported in several cancers [29–31, 37–41], an overall view of ARF6 gene expression in healthy and cancer tissues has been lacking. Further, the public database data on ARF6 expression in the healthy and diseased state is either incomplete or extremely confusing. In this report, we, therefore, analysed the expression of ARF6 at the mRNA level from cancers and corresponding healthy controls from 17 different tissues. We further evaluated ARF6 protein expression in EAC, using a tissue microarray. *ARF6* mRNA expression was found to be at low levels in the adrenal gland, breast, cervix, oesophagus, colon and the endometrium, at moderate levels in the lung, lymphoma, bladder, thyroid, stomach, testis, liver and the highest levels in the kidney, prostate, ovary and pancreas. Moreover, *ARF6* gene expression was substantially increased in oesophageal and lymphoid cancers whereas it was insignificantly decreased in colon cancer. It is worth noting that these findings are subject to limitations of sample size and the heterogeneous nature of certain cancers (such as breast cancer and Oesophageal adenocarcinoma [EAC]). Further assessments should be done to validate and expand the differential expression of *ARF6* in healthy and cancer tissues.

The data presented here show low expression of *ARF6* mRNA in the adrenal gland. However, ARF6 has been shown to play an important role in exocytosis in adrenal chromaffin cells expressing ARF6 [21, 42–44]. This suggests that although *ARF6* mRNA expression is relatively low, there is still enough ARF6 protein produced to fulfil this function. One consideration is that those studies used accumulated chromaffin cells which may express ARF6 at higher levels than the other areas of the adrenal gland. Another reason for this discrepancy could be that the chromaffin cells used in those studies were from bovine as opposed to the whole human adrenal gland used in this study. This may imply the differential expression of ARF6 between humans and cows.

ARF6 is expressed highly in the more invasive and migratory cell lines and, whilst ARF1 was additionally shown to be implicated in the invasion, migration was revealed to be a function of ARF6 alone, amongst the ARFs [30, 31, 45–47]. Although glioma tissue was not included in this study, it has been previously used to correlate increased ARF6 expression with high-grade tumour tissues and cell lines [29]. In these cells, ARF6 is required for the epidermal growth factor (EGF) induced cell proliferation [29]. It has been suggested that the increased presence of ARF6 may increase the rate of membrane trafficking of the EGF receptor (EGFR) and therefore increase intracellular signalling through this receptor [29]. Oesophageal, cervix, and lymphoid cancers showed significant upregulation of *ARF6* mRNA expression in this study, therefore the discussed roles of ARF6 in glioma may be pertinent to these cancers.

Oesophageal cancer is divided into two molecularly distinct diseases (squamous cell carcinoma [ESCC] and EAC), which also differ in prognosis and therapeutic strategies [48]. Findings in the present study are consistent with a cancer genome atlas study, which suggested that these two oesophageal subtypes significantly differ in many signalling pathways. The ARF6 signalling pathway, which is associated with cell motility, is moderately increased in EAC in contrast to ESCC [49]. ARF6 has also been implicated in promoting cell invasion of EAC [50]. An important finding to emerge in this study is the significant upregulation of ARF6 in EAC, indicating the possibility of ARF6 and its signalling events as molecular signatures of EAC. However, a lot more research work needs to be done to suggest that ARF6 could be a prognostic biomarker for EAC, which current promising biomarkers include COX-2, MET and LC3B [51]. Future research should concentrate on understanding the molecular mechanism of high *ARF6* mRNA expression and differences in *ARF6* mRNA expression between EAC and ESCC, which has greater implications for therapeutic strategies. Although *ARF6* mRNA expression was shown in this study to be unaltered in breast and pancreatic cancers, an increase in ARF6 protein levels in these cancers has been reported previously [36, 52], indicating regulation of ARF6 expression at the translational level. Consistent with this, it has been shown recently that *ARF6* mRNA contains a G-quadruplex structure at the 5’-untranslated region and eukaryotic translational initiation factor (eIF)4A controls ARF6 protein expression by binding to the G-quadruplex structure of ARF6 mRNA [52].

In addition to ARF6, ARF regulators (ARF GEFs and GAPs) have also been associated with the invasion and metastasis of cancer cells [6]. GEP100/BRAG2 (an ARF6 GEF) expression is higher in invasive breast carcinoma, and its recruitment to EGFR in breast cancer cells results in ARF6 activation and stimulation of cell invasion [53]. Increased expression of another ARF6 GEF, EFA6A, has been shown in glioma and linked to promoting invasiveness [53], while EFA6B (in breast cancer) and EFA6R (in ovarian cancer) have been shown to play possible roles as tumour suppressors [54, 55]. ARF6 (GAPs), such as SMAP1, AMAP1 and GIT1, are also linked to cancer progression [56]. In summary, among the ARF family of small GTPases, ARF6 is uniquely involved in many cancer types and sub-types. In this study, we showed differential expression of *ARF6* mRNA in 17 different cancer tissues with a particular focus on EAC–given a significant upregulation of ARF6 in this cancer.

## Supporting information

S1 Fig Immunoblot analysis of the specificity of the ARF6 antibody. (PDF)

S2 Fig DNA sequencing of the PCR product in Fig 1D and alignment of the sequence with Arf6 gene sequence. (PDF)
