# Supplementary material for: ADP-ribosylation factor 6 expression increase in oesophageal adenocarcinoma suggests a potential biomarker role for it
Source: PLoS One. 2022 Feb 10;17(2):e0263845. doi: 10.1371/journal.pone.0263845 (PMC8830706; doi:10.1371/journal.pone.0263845)
Supplement: S1 Fig — (PDF) [file pone.0263845.s001.pdf]

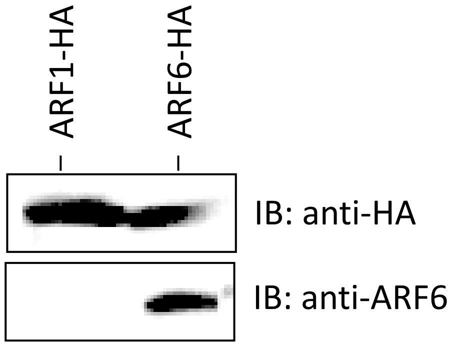

Figure S1 Immunoblot analysis of the specificity of the ARF6 antibody. The lysates of COS7 cells expressing HA-tagged ARF1 and ARF6 were immunoblotted (IB) using the anti-ARF6 antibody 3A-1 mouse monoclonal antibody (Santa Cruz Biotech., US, 1 in 200 dilution) or an anti-HA mouse monoclonal antibody (Biolegend, UK, 1 in 1000 dilution). The ARF6 antibody recognised only ARF6 whereas the HA antibody detected both the HA-tagged ARFs.
