# Supplementary material for: ADP-ribosylation factor 6 expression increase in oesophageal adenocarcinoma suggests a potential biomarker role for it
Source: PLoS One. 2022 Feb 10;17(2):e0263845. doi: 10.1371/journal.pone.0263845 (PMC8830706; doi:10.1371/journal.pone.0263845)
Supplement: S2 Fig — (PDF) [file pone.0263845.s002.pdf]

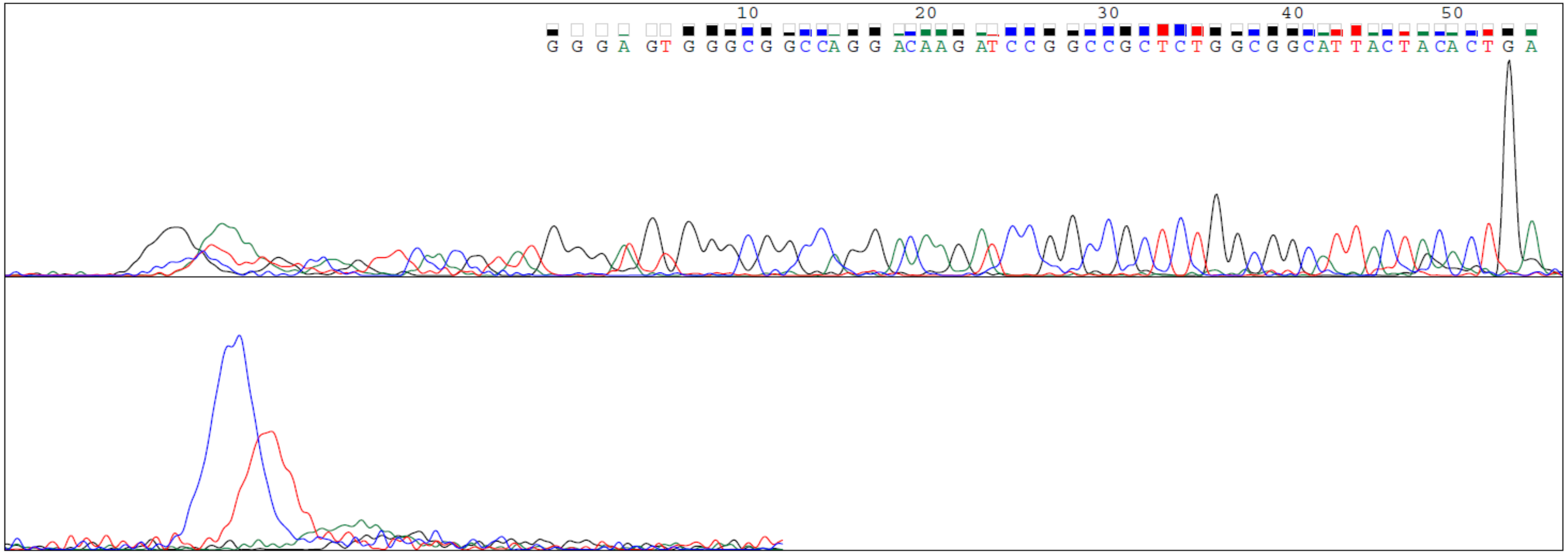

Homo sapiens ADP ribosylation factor 6 (ARF6), mRNA
Sequence ID: **NM_001663.3** Length: 3939 Number of Matches: 1
Range 1: 811 to 859

Score	Expect	Identities	Gaps	Strand	Frame
91.6 bits(49)	4e-17()	49/49(100%)	0/49(0%)	Plus/Plus	

Features:

```
Query  1    GTGGGCGGCCAGGACAAGATCCGGCCGCTCTGGCGGCATTACTACACTG  49
            |||||||||||||||||||||||||||||||||||||||||||||||||
Sbjct  811  GTGGGCGGCCAGGACAAGATCCGGCCGCTCTGGCGGCATTACTACACTG  859
```

Figure S2 DNA sequencing of the PCR product in Fig 1D and alignment of the sequence with Arf6 gene sequence.
